# A New Carbohydrate Food Quality Scoring System to Reflect Dietary Guidelines: An Expert Panel Report

**DOI:** 10.3390/nu14071485

**Published:** 2022-04-02

**Authors:** Adam Drewnowski, Matthieu Maillot, Yanni Papanikolaou, Julie Miller Jones, Judith Rodriguez, Joanne Slavin, Siddhartha S. Angadi, Kevin B. Comerford

**Affiliations:** 1 Center for Public Health Nutrition, University of Washington, Seattle, WA 98195, USA; adrewnow@fredhutch.org; 2 MS-Nutrition, Faculté de Médecine La Timone, CEDEX 5, 13385 Marseille, France; matthieu.maillot@ms-nutrition.com; 3 Nutritional Strategies Inc., Nutrition Research & Regulatory Affairs, Paris, ON N3L 0A3, Canada; papanikolaou.yanni@gmail.com; 4 Emerita, Department of Nutrition and Exercise Science, St. Catherine University, St. Paul, MN 55105, USA; juliemjones@comcast.net; 5 Department of Nutrition & Dietetics, Brooks College of Health, University of North Florida, Jacksonville, FL 32224, USA; jrodrigu@comcast.net; 6 Department of Food Science and Nutrition, University of Minnesota, St. Paul, MN 55108, USA; jslavin@umn.edu; 7 School of Education and Human Development, University of Virginia, Charlottesville, VA 22904, USA; ssa2w@virginia.edu; 8 OMNI Nutrition Science, Davis, CA 95616, USA

**Keywords:** carbohydrate foods, nutrient profiling, fiber, free sugars, whole grain, sodium, potassium, Dietary Guidelines for Americans

## Abstract

Existing metrics of carbohydrate food quality have been based, for the most part, on favorable fiber- and free sugar-to-carbohydrate ratios. In these metrics, higher nutritional quality carbohydrate foods are defined as those with >10% fiber and <10% free sugar per 100 g carbohydrate. Although fiber- and sugar-based metrics may help to differentiate the nutritional quality of various types of grain products, they may not aptly capture the nutritional quality of other healthy carbohydrate foods, including beans, legumes, vegetables, and fruits. Carbohydrate food quality metrics need to be applicable across these diverse food groups. This report introduces a new carbohydrate food scoring system known as a Carbohydrate Food Quality Score (CFQS), which supplements the fiber and free sugar components of previous metrics with additional dietary components of public health concern (e.g., sodium, potassium, and whole grains) as identified by the Dietary Guidelines for Americans. Two CFQS models are developed and tested in this study: one that includes four dietary components (CFQS-4: fiber, free sugars, sodium, potassium) and one that considers five dietary components (CFQS-5: fiber, free sugars, sodium, potassium, and whole grains). These models are applied to 2596 carbohydrate foods in the Food and Nutrient Database for Dietary Studies (FNDDS) 2017–2018. Consistent with past studies, the new carbohydrate food scoring system places large percentages of beans, vegetables, and fruits among the top scoring carbohydrate foods. The whole grain component, which only applies to grain foods (*N* = 1561), identifies ready-to-eat cereals, oatmeal, other cooked cereals, and selected whole grain breads and crackers as higher-quality carbohydrate foods. The new carbohydrate food scoring system shows a high correlation with the Nutrient Rich Food (NRF9.3) index and the Nutri-Score. Metrics of carbohydrate food quality that incorporate whole grains, potassium, and sodium, in addition to sugar and fiber, are strategically aligned with multiple 2020–2025 dietary recommendations and may therefore help with the implementation of present and future dietary guidelines.

## 1. Introduction

Carbohydrate foods (CFs), including grains, starchy roots and tubers, legumes, vegetables, and fruit, account for more than half of the dietary energy in the global food supply [1]. Research suggests that the quality of CFs can impact overall diet quality and health outcomes [2]. However, at present there are no standardized methods for assessing CF quality. Metrics of carbohydrate quality have recently focused on fiber and free sugars—specifically, on fiber-to-carbohydrate ratios and free sugar-to-carbohydrate ratios [3,4]. Although free sugars and fiber are certainly important dietary components to consider when assessing CF quality, the diversity of CF sources may require novel metrics that go beyond sugar and fiber to incorporate other nutrients of public health concern, such as sodium, potassium, and/or other recommended dietary components, such as whole grains.

Methods to assess the quality of CFs should reflect current dietary guidance. Sodium and potassium are both found in CFs and are consumed out of balance with recommendations from the Dietary Guidelines for Americans (DGA) [5]. Although sodium is considered a nutrient to limit, potassium has been identified as a shortfall nutrient in the 2020–2025 DGA [5]. The inclusion of these micronutrients in a CF quality metric could be useful for improving both CF selection and overall diet quality. Reducing sodium content of certain CFs, such as bread, has been a priority for regulatory agencies and for food manufacturers. The US Food and Drug Administration (FDA) recently released guidance for the industry on reducing the sodium content of processed foods, including CFs [6]. Among important CF sources of dietary potassium are starchy roots and tubers, legumes, vegetables, and fruits [7,8].

In addition to nutrients of public health concern, the 2020–2025 DGA emphasizes the importance of including other dietary components, such as whole grains, in the everyday diet [5]. The Healthy Eating Index (HEI 2015), which is a measure of diet quality that aligns with each successive DGA, assigns positive points to whole grains and negative points to refined grains (although the HEI 2015 does acknowledge that reasonable levels of refined grains (≤1.8 oz equivalents/1000 kcal) can fit into a healthy dietary pattern) [9]. This approach aligns with the recommendations of the World Health Organization (WHO) and roughly half of food-based dietary guidelines (FBDGs) from around the world, all of which promote the consumption of whole grains [5,10,11]. Developing a new CF quality scoring system that includes whole grains, potassium, and sodium alongside free sugars and fiber would not only be more inclusive of the range of CFs but could also help in the implementation of dietary guidance [12].

This report builds on an earlier Quality Carbohydrate Coalition-Scientific Advisory Council (QCC-SAC) publication from July 2021 [13]. That publication reviewed the current state of CF quality metrics and established several principles for developing a new scoring system for determining CF quality. The present research report advances this work by (1) introducing a more broadly applicable carbohydrate food quality scoring system, known as a Carbohydrate Food Quality Score (CFQS), and by (2) comparing two CFQS models to other measures of nutrient density (i.e., Nutrient-Rich Food (NRF) index, Nutri-Score) and carbohydrate quality (i.e., 10:1:1 carbohydrate:fiber:free sugar model of Liu et al.) [4].

## 2. Materials and Methods

### 2.1. Defining and Identifying Carbohydrate Foods

Following past studies [4], this analysis investigated selected CFs that are most relevant to the 2020–2025 DGA and are of interest to manufacturers of carbohydrate-containing food products. Only solid foods with ≥40% energy from carbohydrate (as measured by 100 g dry weight) were included. Based on the What We Eat in America (WWEIA) classification codes, categories selected from the grains and snacks and sweets groups were: cooked grains, breads and rolls, quick breads, ready-to-eat (RTE) cereals, cooked cereals, savory snacks, crackers, snack/meal bars, sweet bakery products, candy, and other desserts. The analysis also included beans, peas and legumes, vegetables (including white potatoes), and whole fruits. Relevant grain-based mixed dishes were included as well [4]. Fruit juices and sugar-sweetened beverages were not included. Milk and dairy products, other than some sweetened dairy desserts, were not viewed as primary carbohydrate sources and were excluded from the analysis. The analysis also excluded baby foods and infant formulas, non-reconstituted nutrition powders, and items not classified as foods. The analytical database used in the present analysis comprises 2596 foods, including 1561 grain foods. Candy and desserts were not considered to be grain foods.

### 2.2. Carbohydrate Foods Listed in Food and Nutrient Database for Dietary Studies (FNDDS)

The Food and Nutrient Database for Dietary Studies (FNDDS) 2017–2018 supplied by the US Department of Agriculture (USDA) was utilized to calculate the energy and nutrient content of foods for this study [14]. The complete FNDDS 2017–2018 database contains information on 7083 items, categorized using WWEIA coding schemes. One-digit codes identify 9 major food groups: grains, snacks and sweets, beans and nuts, vegetables, fruits, milk, meat, eggs, and fats and oils. Two-digit WWEIA codes identify 53 smaller food subgroups. Foods in the grains group are separated into cooked grains, breads, quick breads, RTE cereals, and cooked cereals. Four-digit WWEIA codes identify 138 food categories. For example, savory snacks, coded under snacks and sweets, are separated into potato chips, tortilla and other chips, popcorn, and pretzels and snack mixes. Sweet bakery products, also coded under snacks and sweets, are separated into cakes and pies, cookies and brownies, and doughnuts, pastries, and sweet rolls. The eight-digit WWEIA codes correspond to individual foods. The FNDDS lists the nutrient composition of foods as consumed by National Health and Nutrition Examination Survey (NHANES) participants and not as purchased in the store. As such, the FNDDS database has been used in many past studies to assess the overall quality of the American diet. The version of FNDDS used for this study corresponds to the NHANES 2017–2018. The FNDDS was merged with the Food Patterns Equivalents Database (FPED), which provides the content of whole grains and other food components. Free sugars are defined as added sugars; sugars from 100% fruit juice; sugars in sweetened beverages, jams, and jellies; and honey, sugars, and syrups. All databases are maintained by the USDA and are available online.

### 2.3. Selection of Dietary Components for Inclusion in Carbohydrate Food Quality Scores

The components deemed to be the most relevant to the development of a carbohydrate food quality scoring system are energy density (kcal/100 g), fiber, free sugar, sodium, and potassium. The whole grain content of foods, another important component of carbohydrate quality, was obtained from the FPEDs database and converted to g/100 g. Given that CFs can vary in energy density (i.e., moisture content), food dry weight and carbohydrate dry weight were calculated as derived variables.

### 2.4. Building on Existing Metrics of Carbohydrate Quality

Fiber and free sugar content of CFs are commonly used nutrient-based metrics for assessing carbohydrate quality [4,15]. This analysis is built on earlier models of CF quality that were based on 10% cutoffs by weight for sugar and fiber and which had been used to assess diet quality in relation to cardiovascular disease risk [16,17]. In recent years, Liu et al. [4] expanded the original 10:1 model by including free sugars, also in the 10:1 ratio, with a passing score for a ‘higher nutritional quality carbohydrate-rich food’ defined as ≥1 g of dietary fiber and <1 g of free sugars per 10 g of carbohydrate. The 10:1:1 scheme was selected based on the recommended consumption of 50% of energy from carbohydrates but only 5% from free sugars [5,18]. The use of ratios in CF quality metrics was justified by their simplicity and by the widely available data on the carbohydrate, sugar, and fiber content of foods, which are all listed on the Nutrition Facts Panel.

### 2.5. New Models of Carbohydrate Food Quality

Nutrient profiling (NP) systems are used to characterize the nutritional value of foods and can be used to assist in the construction of healthy food patterns as recommended by dietary guidelines. The current DGA recommends reducing free (or added) sugar and sodium, while increasing whole grains, fiber, and potassium. All these elements are of interest when assessing overall CF nutritional quality [5,13,19]. The elements of the new carbohydrate food scoring system are listed in Table 1 below. The point system follows the approach of Liu et al. [4]. The 10% cutoffs for fiber and free sugars relative to carbohydrates (i.e., 10:1:1) are the same as previously used, with the exception that they are scaled per 100 g portion for CFQS models. The cutoff level for sodium is based on <600 mg sodium/100 g dry weight. The cutoff for potassium is >300 mg/100 g dry weight. These values roughly correspond to the median values for each nutrient in the FNDDS. Similar median splits are used in metrics of adherence to Mediterranean diets, allowing for maximum discriminating power [20]. The whole grain cutoff of ≥25% whole grain dry weight is based on past studies that awarded a passing score to foods containing ≥25% whole grain on a dry-weight basis [21,22]. Current US regulations state that foods containing at least 25% whole grain on a dry-weight basis may make a front-of-pack claim. For foods to be classified as whole grain, at least 50% by dry weight is required. 

Based on the cutoff values and scoring system presented in Table 1, two models for assessing CF quality were developed (Table 2). One model is designated the CFQS-4 model since it is based on 4 component scores (fiber, free sugar, sodium, and potassium), and the other is designated the CFQS-5 model since it adds whole grains as a 5th component score. For the CFQS-4 model, CFs are eligible to score up to 4 points (1 point for each dietary component measured), with the CFs scoring 4 points being classified as “higher-quality”. For the CFQS-5 model, CFs are eligible to score up to 5 points (1 point for each dietary component being measured), with CFs scoring 4 or 5 points being classified as “higher-quality”.

### 2.6. Statistical Analysis

For all carbohydrate foods, the numbers and proportions of items achieving top scores for each metric were assessed. Kappa statistics were used to assess the agreement between the three composite metrics of carbohydrate quality. Comparisons of CFQS with other nutrient density metrics were based on Pearson correlations.

## 3. Results

### 3.1. Identifying Carbohydrate Foods in FNDDS 2017–2018

**Figure 1:**
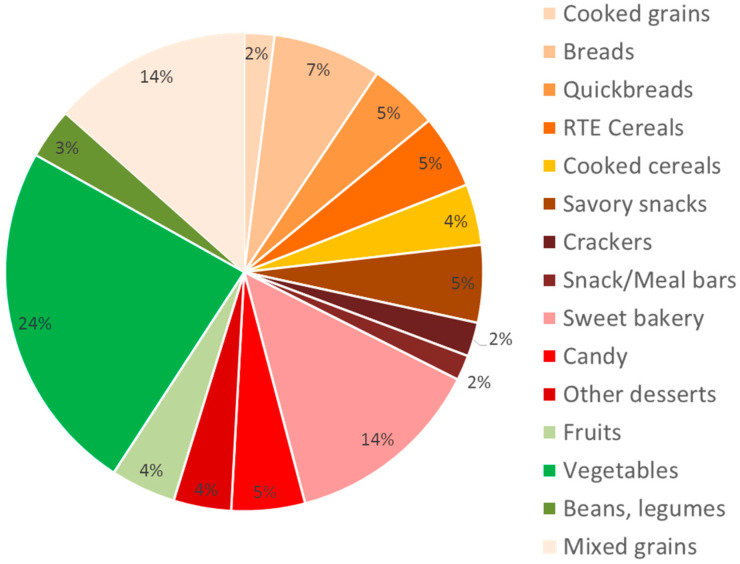
Distribution of Carbohydrate Foods in the Food and Nutrient Database for Dietary Studies (FNDDS) (*N* = 2596).

The present analytical sample comprises 2596 FNDDS foods (Figure 1 and Table 3). The sample includes 362 mixed-grain dishes (14%), 357 (14%) sweet bakery products; 198 (7%) breads, rolls, and tortillas; 134 (5%) cold RTE cereals; 111 (4%) cooked cereals; 140 (5%) savory snacks; 126 (5%) quick breads and bread products; 55 (2%) cooked grains; 62 (2%) crackers; 46 (2%) snack/meal bars; 130 (5%) candy products; 105 (4%) other desserts; 117 (4%) fruits; 561 (24%) vegetables, including white potatoes; and 92 (3%) beans and legumes. For analyses specific to whole grain foods, the sample is restricted to 1561 CFs in the grains and snacks and sweets categories, and it excludes potato chips, chocolate and non-chocolate candy, and “other desserts” which generally comprise non-grain carbohydrates, such as ice cream and jellies.

The distribution by food groups is shown in Figure 1. The present total sample of 2596 foods is broadly similar to that of Liu et al. [4], who identified a total of 2208 carbohydrate-rich processed products in FNDDS, namely 386 (17.5%) sweet bakery products; 206 (9.3%) breads, rolls, and tortillas; 198 (9.0%) cold cereals; 197 (8.9%) cooked cereals; 177 (8.0%) savory snacks; 143 (6.5%) quick breads and bread products; 82 (3.7%) cooked grains; 80 (3.6%) crackers; 46 (2.1%) snack/meal bars; 19 (0.86%) smoothies and grain drinks; 55 (2.5%) baby foods; and 619 (28.0%) mixed dishes. Since Liu et al. [4] focused on processed products, they did not consider fruits, vegetables, and legumes, which are generally thought to provide higher-quality carbohydrates and to be integral components of healthy dietary patterns [5]. The present analysis excludes most mixed dishes, which contain significant amounts of both protein and fat, making them ill-suited for inclusion in a carbohydrate category-specific food scoring metric.

### 3.2. Components of the Carbohydrate Food Quality Scoring System

#### 3.2.1. Component Scores

The component score for fiber shown in Table 3 uses the same criteria as the 10:1 carb:fiber model used by Liu et al. [4]. Nearly all beans and legumes, and most vegetables and fruit, contain ≥10 g of fiber per 100 g of carbohydrate. Foods with lower fiber content are primarily sweet bakery goods, other desserts, quick breads, and candy. The component score for free sugar is a separate element of the 10:1:1 carb:fiber:free sugar model of Liu et al. [4]. Our analyses show that most beans and legumes, vegetables, fruit, savory snacks, and cooked grains earn this point, as they contain little or no free sugar (Table 3). Foods with higher sugar content are primarily sweet bakery goods, candy, other desserts, snack/meal bars, and most RTE cold cereals.

Component scores for sodium, potassium, and whole grains are additional elements that are included in the CFQS models. Sodium and potassium have not traditionally been considered in measures of carbohydrate quality, whereas whole grains have been included in previous carbohydrate quality metrics (e.g., whole grains:total grains ratio) [23] and have been used to assess the overall carbohydrate quality of a dietary pattern [24,25]. The analysis shows that many vegetables (as prepared and consumed) contain more than 600 mg sodium/100 g dry weight and do not benefit from the sodium point score (Table 3). Breads tend to be high in sodium, whereas cooked cereals do not. Many food items that are high in free sugars are low in sodium. Component scores for potassium perform as expected. The CFs that are most likely to score potassium points are beans, vegetables (including white potatoes), and fruits, and those least likely to score potassium points are cooked cereals, snacks/meal bars, and other desserts.

Whole grain content is calculated per dry weight, following prior studies [20,21]. Most RTE cereals score a whole grain point for having ≥25% whole grains, as do about half of breads, cooked cereals, crackers, and savory snacks (Table 3). Most sweet bakery goods, other desserts, snack/meal bars, and candy do not score a whole grain point. Since beans, legumes, vegetables, and fruit do not contain whole grains, they are ineligible for scoring a whole grain point. As a result, only 436 foods out of 2596 are awarded a whole grain point in the analysis.

#### 3.2.2. Carbohydrate Food Quality Scores (CFQS)

Based on the scores presented in Table 3, higher-quality CFs represent 29.2% of the total in the 10:1:1 model, 10.5% of foods in the CFQS-4 model, and 15.3% of foods in the CFQS-5 model. Kappa values indicate a fair correspondence between the 10:1:1 model and the CFQS-4 model (kappa 0.455) as well as the CFQS-5 model (kappa 0.464). The correspondence between the two CFQS models is much better: kappa 0.839. All values are significant (*p* < 0.001).

**Figure 2:**
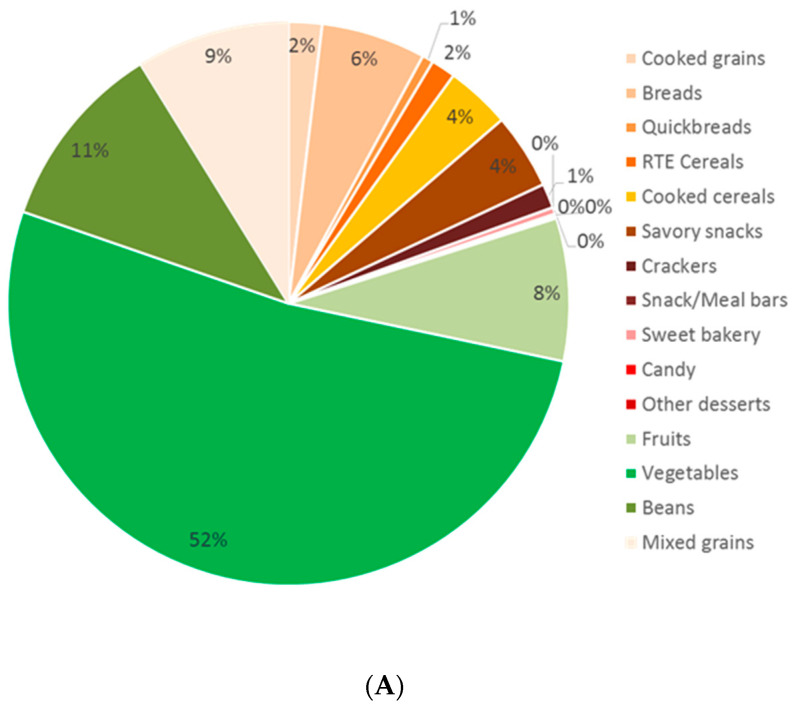

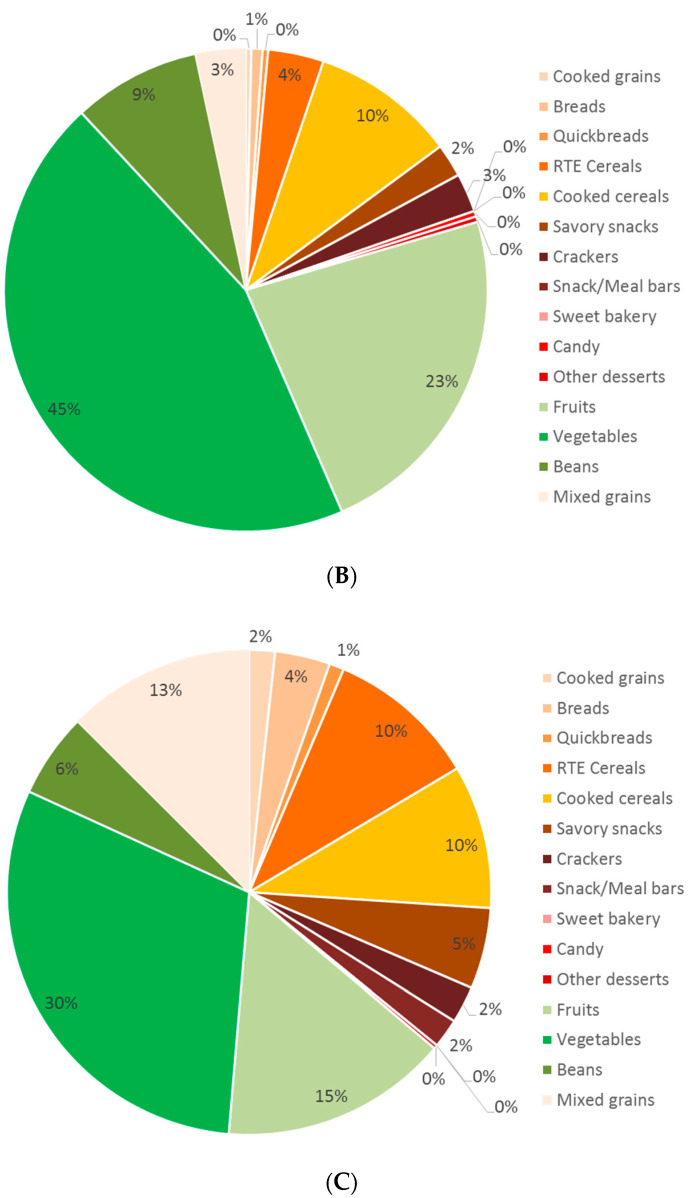
Distribution of higher-quality carbohydrate foods by food group. The three models are: (**A**) 10:1:1 carb:fiber:free sugar; (**B**) CFQS-4: comprised of fiber, free sugar, sodium, and potassium, and (**C**) CFQS-5: comprised of fiber, free sugar, sodium, potassium, and whole grains (*N* = 2596).

The distribution of CFQS by food category is shown in Figure 2, which includes scores based on fiber and free sugar (Figure 2A: the 10:1:1 score of Liu et al. 2020 [4]); the CFQS-4 model based on fiber, free sugar, sodium, and potassium (Figure 2B); and the CFQS-5 model based on fiber, free sugar, sodium, potassium, and whole grains (Figure 2C). 

Figure 2 shows that all three CF quality models favor fruit, vegetables (including white potatoes), and beans. Figure 2A (10:1:1) shows that a scoring system based on two components (fiber and free sugars) results in vegetables (52%), beans (11%), mixed grains (9%), and fruits (8%) being heavily represented in the top scoring category. Figure 2B (CFQS-4) shows a similar distribution for vegetables (45%) and beans (9%) when using a four-component score based on fiber, free sugar, sodium, and potassium. The inclusion of sodium and potassium in this scoring system nearly triples the percentage of fruits (23%), more than doubles the cooked cereals (10%), and majorly reduces the number of mixed grain dishes (3%) considered to be higher-quality CFs. The addition of a whole grain point shifts the distribution, as shown in Figure 2C (CFQS-5). When compared to the 10:1:1 and CFQS-4 models, the CFQS-5 classifies several more mixed grain dishes (13%), RTE cereals (10%), and savory snacks (5%) as higher-quality CFs. Vegetables (30%) and fruits (15%) are not eligible to benefit from the whole grains score but still account for a large proportion of higher-quality CFs in the CFQS-5 model. Regarding grain foods, the main differences between the CFQS-4 and CFQS-5 models are the increased percentages of RTE cereals (4% vs. 10%, respectively) and savory snacks (2% vs. 5%, respectively) being classified as higher-quality CFs.

**Figure 3:**
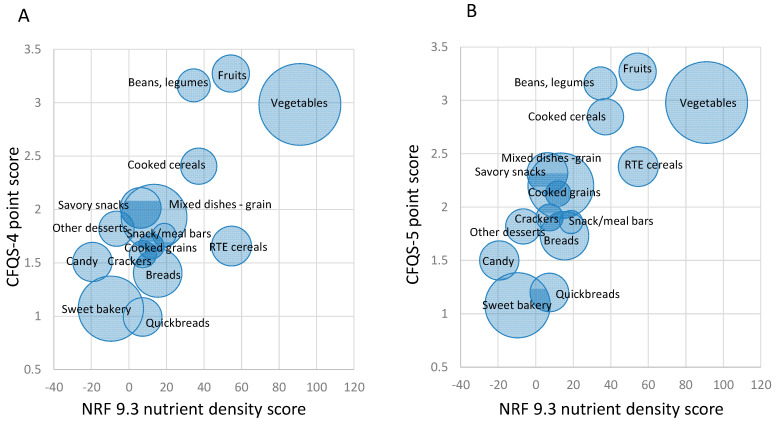
Relation between Carbohydrate Food Quality Scores (CFQS) and NRF9.3. *N* = 2596. (**A**) CFQS-4 vs. NRF9.3 Scores for 15 Carbohydrate Food Categories (**B**) CFQS-5 vs. NRF9.3 Scores for 15 Carbohydrate Food Categories (The size of the circles represents the number of higher-quality carbohydrate foods in each food category).

Figure 3 shows the relation between the CFQS model scores and the Nutrient-Rich Foods Index 9.3 (NRF9.3) score for 2596 foods. The size of the circles denote the number of higher-quality items per food category. Both CFQS-4 and CFQS-5 models appear to score beans, legumes, and fruits as higher-quality CFs relative to the NRF9.3. The addition of the whole grain scoring component in the CFQS-5 (Figure 3B) improves the scores of various grains and the snacks and sweets categories relative to beans, legumes, vegetables, and fruit. Regarding grain foods, several foods from the mixed grain dishes, cooked cereals, cooked grains, breads, quick breads, RTE cereals, savory snacks, crackers, and snack/meals bars categories score 1 point higher in the whole grain inclusive model, in turn bringing the entire category up, whereas the sweet bakery products category stays the same. The correlation between the CFQS-4 and NRF9.3 is 0.510 (*p* < 0.0001), and the correlation between the CFQS-5 and NRF9.3 is 0.477 (*p* < 0.001). Both CFQS metrics are strongly related to the well-established NRF9.3.

### 3.3. Carbohydrate Food Quality Score Applied to Grains, Snacks and Sweets

**Figure 4:**
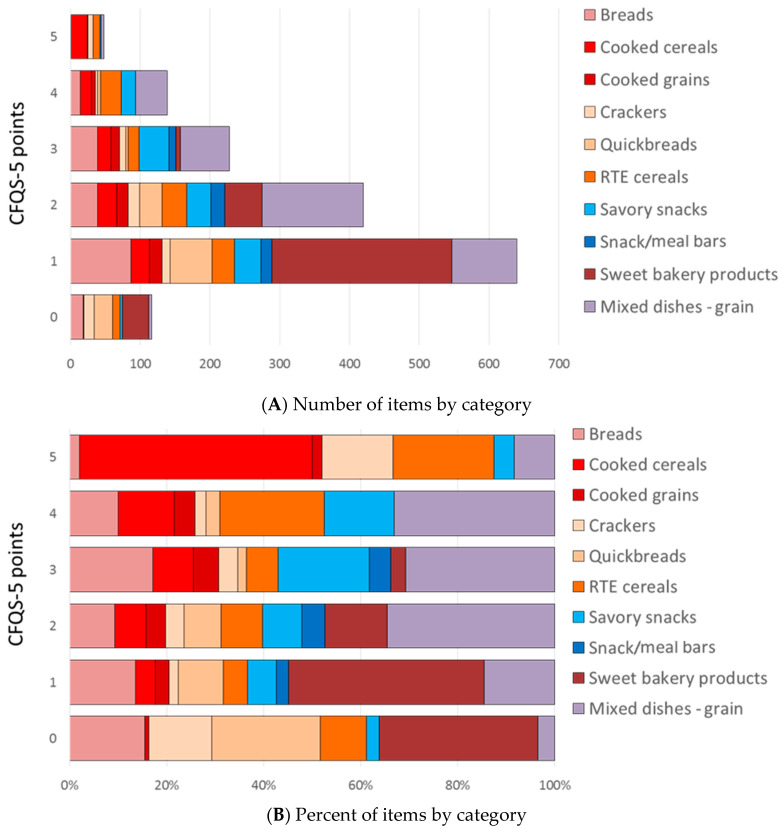
Distribution of CFQS-5 scores for the grain group (*N* = 1561) shown as point values (**A**) and as percentages (**B**). Figure 4A shows absolute values by food category, whereas Figure 4B shows the relative percent contribution from each food category to each point score.

The whole grains component does not alter scores from fruits, vegetables, or beans, since those items do not contain whole grains. However, the CFQS-5 model does allow for finer discrimination of carbohydrate quality within the grains and the snacks and sweets food categories. These data are shown in Figure 4. For these calculations, beans and legumes, vegetables, and fruit are dropped. Likewise, chocolate and non-chocolate candy, ice cream and gelatins, and other desserts are also not included in the calculations. The analytic database comprises 1561 grain food items. Figure 4 shows that most sweet bakery goods score no more than 2 points in the CFQS-5 model. Mixed grain dishes, breads, and savory snacks make up the largest portion of the 3-point scores. Mixed grain dishes and RTE cereals make up the largest portion of the 4-point scores, whereas cooked cereals make up the largest portion of the 5-point scores.

### 3.4. Relative Validity of the CFQS-5 as Compared to the NRF9.3 and Nutri-Score

**Figure 5:**
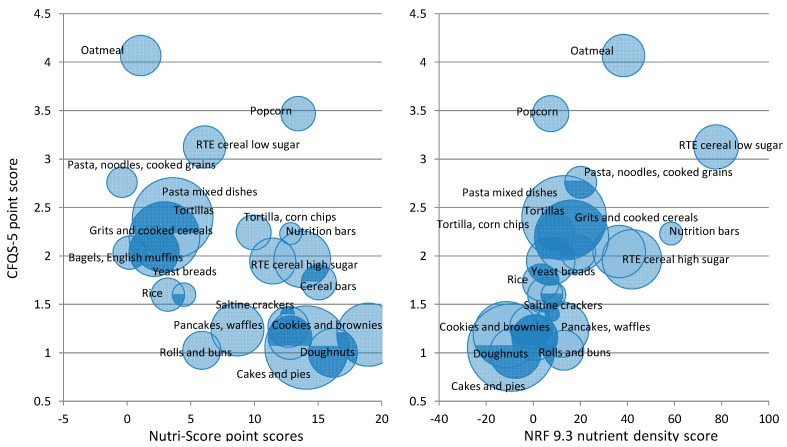
Relation between CFQS-5 Model, Nutri-Score and NRF9.3. (The size of the circles represents the number of higher-quality carbohydrate foods in each food category).

Further validation of the CFQS-5 model, when only evaluating grain foods (*N* = 1561), is compared against NRF9.3 and Nutri-Score in Figure 5. For grain foods, the CFQS-5 model correlates with NRF9.3 (r = 0.458; *p* < 0.001) and Nutri-Score (r = −0.447; *p* < 0.001). NRF9.3 and Nutri-Score are inversely correlated for this category of food products (r = −0.476; *p* < 0.001). Nutri-Score is very highly correlated with the energy density of foods (r = 0.697; *p* < 0.001), whereas the CFQS-5 model is not (r = −0.127; *p* < 0.001).

## 4. Discussion

Carbohydrate quality metrics based on ≥10% fiber content per 100 g carbohydrate (10:1 carb/fiber ratio) have been used for more than a decade [17] to identify higher nutritional quality CFs and higher quality diets [4,26]. Recently developed CF quality metrics have incorporated a free sugar component (<10% per 100 g carbohydrate) to further differentiate the nutritional quality of CFs [4]. The 10:1:1 carb:fiber:free sugar model of Liu et al. [4] is consistent with dietary guidelines. Both fiber and free sugars are considered macronutrients of public health concern in the 2020–2025 DGA with the importance of increasing dietary fiber and reducing free sugars being emphasized for achieving healthy dietary patterns [5].

The CFQS-5 model adds three components to the 10:1:1 model of Liu et al. [4] to better align with DGA recommendations. These are included since the DGA features whole grains as an important component of carbohydrate food quality. Additionally, sodium reduction in processed foods, including grain-based foods, is a priority for both the DGA and the FDA. Lastly, potassium has been identified as a shortfall nutrient, and the potassium content of foods is required to be listed on FDA-approved food labels. The present CFQS models are designated based on their number of components; the CFQS-4 model includes four components: fiber (F), free sugar (S), sodium (Na), and potassium (K), whereas the CFQS-5 model adds whole grains, being inclusive of five dietary components.

In contrast to earlier studies [4,26], our analytical sample is not limited to grain foods, and instead focuses on 2596 CFs (defined as solid foods which are ≥40% carbohydrate by dry weight) from multiple foods groups listed in the FNDDS 2017–2018. The present sample includes 1561 grain foods (inclusive of 10 different food categories), along with 561 vegetables (including white potatoes), 117 fruits, 92 beans and legumes, 105 non-grain desserts, and 130 candies. Most of the non-grain foods in the sample come from food groups that are generally recognized as containing higher-quality carbohydrates (i.e., vegetables, fruits, and beans and legumes), and candy, a “low-quality” source of carbohydrates, is also included in the sample for internal validation purposes.

In general, most fruits, beans and legumes, and vegetables are classified among higher-quality carbohydrates in the 10:1:1 model and both CFQS models, with nearly all individual vegetables, beans, and legumes considered higher-quality CFs in all models (Table 3). The results also show that, when compared to the 10:1:1 model, the CFQS-4, model, which only considers nutrients of public health concern (fiber, free sugar, sodium, and potassium) and not whole grains, provides similar capabilities for discerning higher-quality CFs from cooked cereals, crackers, RTE cereals, and fruits categories but very different capabilities for discerning higher-quality CFs from the breads, cooked grains, savory snacks, vegetables, and beans and legumes categories (Table 3). In total, 777 (29.1%) of the CFs analyzed are considered higher-quality (received a score of 2 pts) according to the 10:1:1 model, whereas only 272 (10.5%) were considered higher-quality (received a score of 4 pts) according to the CFQS-4 model. As expected, the CFQS-5 model, which also includes a whole grain component and a more lenient scoring system (CFs receiving 4 or 5 points are considered higher-quality), allows for 397 (15.3%) CFs to be considered “higher-quality.” CFs benefitting from the whole grain point are primarily from the categories of breads, cooked cereals, cooked grains, RTE cereals, and savory snacks (Table 3).

Further evaluation of the CFQS-5 model, when applied to grains foods only (*N* = 1561), shows how the various grain-based food categories fare based on absolute point score and relative percentages (Figure 4). Sweet bakery products make up the largest portion of 0-, 1-, and 2-point scores. Scores for mixed grain dishes, breads, quick breads, RTE cereals, cooked grains, crackers, and savory snacks all range from 0 to 5 points, showing considerable variation in the nutritional value and whole grain content of individual food items in these categories. There are 187 high scoring items (CFQS-5 scores of 4 or 5 points); the highest percentages are obtained for cooked cereals (35.1%), RTE cereals (29.9%), savory snacks (20.0%), mixed grain dishes (13.8%), and cooked grains (12.7%). The very highest scores (5 points) are given to various types of popcorn, cooked cereals (primarily oatmeal), low-sugar RTE cereals, and selected whole grain breads and crackers that contain little to no sugar or sodium, and which are high in fiber. A sample list of 132 CFs which have been assessed by both CFQS-4 and CFQS-5 models is provided in the Supplementary Materials section (Table S1).

For the whole sample of CFs (*N* = 2596), we tested the validity of both CFQS models against NRF9.3 (Figure 3). These metrics correlate nicely, as most of the CFQS components (i.e., fiber, sugar, potassium, and sodium) are also components of the NRF9.3, with the main difference being that NRF9.3 includes several additional nutrients in its assessment, and the nutrients are measured in a different manner (i.e., NRF nutrients are measured as a ratio of nutrients to calories (i.e., mg of sodium per 100 kcal of food), whereas CFQS nutrients are measured as ratios to other nutrients (i.e., carb:fiber:free sugar) or in relation to dry weight (i.e., mg or g/100 g food dry weight)). Despite these differences, both CFQS models and the NRF9.3 system place beans, legumes, vegetables, and fruit among higher-quality CFs.

For a subset of the analysis which only contains grain foods (*N* = 1561), we also compared the 5-point CFQS-5 model against the NRF9.3 and Nutri-Score (Figure 5). Similar to the NRF9.3, most of the nutrients of concern (i.e., fiber, sugars, sodium) measured in the CFQS models are also measured in the Nutri-Score system, with the main differences being that Nutri-Score also includes energy, saturated fatty acids, protein, and some food group and healthy oil components. Despite these differences, the CFQS-5 model correlates well with both the NRF9.3 and the Nutri-Score systems when assessing carbohydrate-rich grain foods (Figure 5).

### 4.1. Selection of Components for Carbohydrate Food Quality Scores

#### 4.1.1. Fiber and Free Sugars: Macronutrients of Public Health Concern

In a recent comparison of carbohydrate quality metrics on long-term changes in abdominal obesity [3], the simple 10:1 carb:fiber ratio performed comparably to more complex metrics such as the Carbohydrate Quality Index (CQI), which is a composite score that includes measures of (1) total dietary fiber intake, (2) Glycemic Index (GI), (3) whole grains:total grains ratio, and (4) solid carbohydrate:total carbohydrate ratio. CQI scores for each of the four indicators are assessed by quintiles on a 1 to 5 scale, with a top carbohydrate quality score of 20 points possible. The high score for each indicator is based on being in the top quintile for fiber intake, whole grain:total grain ratio, and solid carbohydrate:total carbohydrate ratio, while being in the lowest quintile of GI. Higher CQI scores have been associated with beneficial outcomes, such as higher nutrient intake [23] and lower risk for abdominal obesity [3,27], and cardiovascular disease [28]. Although both CQI and the 10:1 carb:fiber ratio take into account fiber content (total fiber vs. carb:fiber ratio, respectively), the CQI also takes into account aspects of food groups (i.e., whole grain content), the food matrix (i.e., liquid vs. solid carbohydrates), and physiological impacts on the consumer (i.e., GI). All of these elements may contain valuable information on carbohydrate quality. However, it does not make sense to apply all of the CQI components to our analysis, as our present sample only includes solid foods and excludes liquids, and our analytical protocol focuses on key messaging in the 2020–2025 DGA and food and nutrient attributes currently available in publicly maintained databases, neither of which currently applies to GI. Similarly to the CQI, our CFQS models aim to account for both fiber and whole grain content; however, based on the recent literature [3,4], we choose to assess fiber content in the manner utilized by the 10:1 carb:fiber ratio rather than on a total fiber basis.

In a recent analysis of carbohydrate-rich processed foods, Liu et al. found that the 10:1 ratio was the least restrictive model they tested, with 23.2% of their sample meeting the cutoff criteria, and the 10:1:1 model was the most restrictive model they tested, with only 16.4% of foods tested meeting the cutoff criteria [4]. In our analysis, which includes many non-processed foods, we also found that adding the free sugar component (which includes added sugars, as well as sugars from jams, jellies, honey, and syrups) restricts the sample size and is helpful in more strictly differentiating the quality of CFs in certain food categories (Table 3). For example, nearly all food categories listed in Table 3 show very different percentages of CFs meeting the “higher-quality” criteria, as assessed by the Component Fiber and Free Sugar scores (Table 3). Additionally, when comparing the 10:1 vs. 10:1:1 models (in which our Component Fiber Score in Table 3 is the same as the pre-existing 10:1 ratio), we found that adding the free sugar component to the ratio reduces the number of candies considered “higher-quality” from 8.5% to 0%, RTE cereals from 41.8% to 8.2%, and snack/meal bars from 26.1% to 0%, while negligibly impacting the quality scores for generally considered “higher-quality” CF categories, such as cooked grains, beans and legumes, and vegetables. Therefore, we concluded that fiber and free sugars should both be included as components in our models, as they show independent abilities to identify CF quality. Furthermore, both components are listed as nutrients of public health concern in the 2020–2025 DGA, and they are both required to be listed on government-approved Nutrition Facts Panels [5].

Although fiber and sugar are two of the three major macronutrient components of dietary carbohydrates along with starch, their quantity and ratios only represent a portion of how a CF (or a CF-rich diet) can impact health. Foods are complex entities which generally comprise hundreds to thousands of different compounds. Only focusing on the quantitative aspects of two of these components likely misses or masks other important attributes of CFs. For example, the high carb:fiber ratio of starchy vegetables leads to classification as “lower-quality” CFs in the 10:1:1 scoring model, but if a scoring metric is used that reflects that these foods are also low in sodium, high in potassium, and rich in other health-promoting compounds [13,29,30], then many starchy vegetables may instead be classified as higher-quality CFs.

#### 4.1.2. Sodium and Potassium: Micronutrients of Public Health Concern

The 2020–2025 DGA and other FBDGs from around the world, including those from the WHO, consistently promote dietary patterns with lower sodium (i.e., salt) and higher potassium levels [10,31]. Over the last several decades, processed foods have provided most of the sodium in global diets [32], whereas starchy roots and tubers supply up to 80% of the dietary supplies of potassium in many regions of the world [7]. Food processing methods often remove potassium from foods while adding sodium, further compounding this dietary imbalance [33]. In the US, the top dietary sources of sodium primarily come from processed grains (bread, rolls) and carbohydrate-rich mixed dishes (pizza, sandwiches, soup), and higher levels of potassium are primarily found in fruits, vegetables, legumes, whole grains, and starchy roots and tubers [7,8]. Nearly 90% of US adults exceed the recommended sodium intake, and only 30% meet the adequate intake (AI) for potassium [34].

Due to their complex inter-relationships, these electrolyte minerals are often studied in tandem, with the dietary sodium:potassium ratio being used in research on animal health for nearly a century [35]. A culmination of evidence from human studies shows that a high sodium-to-potassium ratio is associated with hypertension, cardiovascular disease, and kidney disease, and that simple dietary changes to balance out this ratio may reduce these risks [36,37,38,39]. Although the DGA emphasizes the adequate intake of multiple micronutrients of concern related to CF intake (i.e., potassium for underconsumption and sodium for overconsumption) [5], carbohydrate quality metrics rarely consider the role of these micronutrients in CF quality. Given that imbalances in the intake of these two micronutrients account for a significant portion of the US and global disease burden [40,41,42], they should both be included as integral components of CF quality metrics that are aimed at improving the overall quality of dietary patterns. At this time, sodium and potassium threshold values are calculated per dry weight and are approximately equal to the median values for the sampled foods in the database.

#### 4.1.3. Whole Grains: Food Group Component Listed in 2020–2025 DGA Key Messaging

Grain foods supply more than half of the energy in global diets, with most of this energy coming in the form of carbohydrates [43]. In the US, most of the population meets the recommendations for total grain intake, but the ratios and quantities of grain intake are far from recommended, with roughly 74% exceeding the recommended intake for refined grains and 98% not meeting the recommendations for whole grain intake [5]. The overall 2020–2025 DGA recommendation to “make half your grains whole” has been largely unsuccessful. However, considering that a significant portion of the carbohydrates consumed in the US diet come from refined grains, it is important to note that certain refined grains foods, such as those which have been fortified or enriched, provide significant nutritional contributions to US diets. For example, several studies using data from NHANES show that fortified and enriched refined grain products, including select breads, cooked cereals, and RTE breakfast cereals, are associated with improved nutrient intakes and diet quality [44,45].

Compared to refined grains, whole grains tend to contain higher levels of fiber, micronutrients, anti-nutrients, and bioactive compounds associated with health [46]. In essence, the health value of whole grains may come from the combination of multiple beneficial compounds interacting within a largely intact food matrix, rather than just the sum of their nutrients [47]. Individuals who regularly consume whole grains tend to have greater protection against cardiovascular disease, type 2 diabetes, various cancers, and all-cause mortality compared to those who do not [47]. These major health benefits have led many countries to include key messaging in their dietary guidelines, emphasizing whole grain intake over refined grain intake [47,48].

Despite the known health benefits of higher whole grain intake and decades of recommendations in dietary guidelines, whole grain consumption remains low in most countries, majorly contributing to the global disease burden [49,50]. Changes to whole grain policies and promotions are required to incentivize the industry and to help consumers meet recommendations [51]. At present, whole grains are not considered in most NP models [52], impairing the value of these tools to assist in the implementation of dietary guidance. Including whole grain content in NP models may help align food quality metrics (and especially CF metrics) with the most current dietary guidance, in the US and around the world [47].

### 4.2. Limitations, Opportunities, and Next Steps

Although this study improves upon the alignment of CF quality metrics with current DGA guidance, it does have limitations. Firstly, the “quality” of a food can be represented in many different ways (based on nutrient ratios, health effects, sustainability impacts, etc.), most of which are not captured in a simple metric. Many of these qualities (e.g., nutrient content) fall on a spectrum, and although evidence-based estimates are made to determine proper cutoffs for a scoring system, these cutoffs are reasonably subject to challenge and to change. Secondly, key dietary components in CFs that may directly affect carbohydrate metabolism and overall health (e.g., protein and fat content) are not considered in the CFQS due to a lack of databases on the topic, but they may be considered in future iterations as more data become available on nutrient–nutrient interactions. Thirdly, the study analysis only focuses on solid foods and does not include liquid sources of dietary carbohydrates (juices, sugar-sweetened beverages, or various milks and plant-based beverages) that contribute greatly to daily energy and nutrient intakes. Additionally, NP is not a perfect tool, and, as its name implies, it tends to assess food and diet quality almost wholly on nutrient quantities rather than nutrient qualities (e.g., types of dietary fiber or free sugars) or food qualities (e.g., food matrix and processing effects, food group factors). NP models that continue to focus solely on single nutrients are in danger of falling out of step with the current FBDGs [53].

Another limitation of our CFQS models and NP systems in general is that they often weigh each component similarly, whereas the impacts of underconsumption or overconsumption of certain nutrients may outweigh the impacts of others. Most NP models in use are also limited in that they are across-the-board models, in which a single set of nutrient criteria is applied to all foods in the food supply, regardless of their food group or unique dietary contributions. This is being remedied by the development of hybrid NP models that include both nutrients and food groups [47]. These new metrics of nutritional value of individual foods and total diets better align with current FBDGs, which are moving from a traditional focus on nutrients to more food-based factors [53,54]. Our CFQS models are relatively simple, focusing only on those few elements of CF quality which are specific to the evidence-based recommendations provided by the 2020–2025 DGA. By contrast, some recent attempts at NP, such as the Food Compass Score, employ as many as 54 overlapping factors housed within 9 health-related domains [55].

GI is not included as a component in the CFQS models. Although GI is a tool designed specifically for assessing the impacts of CFs, it has several limitations that we have discussed in detail in a previous publication [13]. In summary, GI has shown promise in a clinical setting for predicting glycemic responses in individuals with type 2 diabetes. However, it is not an ideal surrogate measure of overall CF quality for a number of reasons: (1) GI values may vary drastically based on food preparation methods and the timing and presence of other foods in the diet; (2) GI values depend considerably on the biology and behaviors of the person consuming the food, and they have not shown to be an accurate measure of glycemic response over a range of population demographics; (3) GI values only provide data on a person’s blood glucose response, which is just one of many physiological processes in which food intake can impact nutrition and health; and (4) GI values are not currently recognized as a food quality metric in prevailing FBDGs, nor are these values available in publicly funded databases [13].

As the science and guidance on CF quality develops and more data become publicly available, there are likely to be opportunities to expand on these CFQS models. These opportunities include new criteria that assess specific types of dietary fiber (e.g., soluble/insoluble, sourced from fruit/vegetable/cereal products, intrinsic/added, fermentable/non-fermentable), inclusion of bioactive food compounds (e.g., phytochemicals, anti-nutrients, prebiotics), the effects of food matrices and various forms of food processing, as well as enrichment and fortification. All these potential additions add new layers to the understanding of CF quality, however, the present science behind most of these options is underdeveloped and/or absent from inclusion in FBDGs, Nutrition Facts Panels, and publicly available databases. Of the above options for CFQS expansion, the inclusion of profiling for bioactive food components is an ideal next step, as the evidence for the benefits of many of these compounds on food quality and human health are emerging [56,57], and databases focused on the exploration of these types of compounds are becoming more available [58,59,60,61,62,63]. Bioactive food components tend to track with food group designation (e.g., flavonoids in fruits, glucosinolates in vegetables, and lectins and phytates in grains and legumes) [64] and could potentially be used as additional components in CFQS models to differentiate the CF quality of food groups. As a proof-of-concept, the flavonoid content for fruits has been included in NRF9f.3 scores to help capture the full dietary value of fruits in addition to their nutrient content [54], and both flavonoid content and carotenoid content have been included as part of the Food Compass Score for application to all foods and not just to CFs [55]. Interestingly, when the quality of foods is assessed by the Food Compass Score, the fruits, vegetables, and legumes groups are all ranked highest (i.e., most healthful), whereas grains tend to score much lower, even when they are high in fiber, potassium, and whole grains and when they are low in free sugars and sodium. These results show a very different ability between the Food Compass Score and CFQS models to discriminate grain food quality and require further investigation to determine if a 54-attribute scoring system or a 5-attribute scoring system is more useful for helping to align grain food intake with dietary guidelines and public health messaging. As appears now, the Food Compass Score may undervalue higher-quality grain foods, and/or the CFQS models may overvalue them. One problem that needs to be resolved is how to incorporate metrics from different health domains or those that combine both nutrients and dietary ingredients into a cohesive index of overall nutritional value.

## 5. Conclusions

The CFQS models for assessing CF quality aim to operationalize 2020–2025 DGA advice for improving dietary patterns. These NP models were developed using a composite scoring system in which each individual input is strategically aligned with current DGA recommendations. The four nutrient components of the CFQS models are all nutrients of public health concern related to CFs: two for underconsumption (fiber and potassium) and two for overconsumption (free sugars and sodium). The more complex model (CFQS-5) is a hybridized model which also includes a whole grain component to align the scoring system with DGA recommendations on grain food intake. Food group distinctions in NP models, such as the inclusion of whole grain content, may prove valuable for helping newer models to better discriminate the quality of grain foods when compared to previous metrics, while also helping to identify the top-quality CFs from non-grain food groups (i.e., fruits, vegetables, and beans and legumes). In addition to food group factors, other important components to consider for expanding these CFQS models as more data become available are the type and degree of processing a food undergoes, aspects of a food’s bioactive composition, and whether the food has been fortified or enriched. Additional topics that should be addressed to move this new scoring system forward are its potential integration into current national nutrition and health policies and programs, and the development of user-friendly tools for multiple stakeholders, e.g., government, industry, and consumers.

## Figures and Tables

**Table 1:** Carbohydrate Food Quality Score (CFQS) Components.

Components	Component Scores	Score Range
Fiber	1 point if fiber ≥ 10 g/100 g carb portion; else 0 points	0 to 1
Free Sugar	1 point if free sugar < 10 g/100 g carb portion; else 0 points	0 to 1
Sodium	1 point if Na < 600 mg/100 g dry weight; else 0 points	0 to 1
Potassium	1 point if K > 300 mg/100 g dry weight; else 0 points	0 to 1
Whole Grains	1 point if whole grains ≥ 25 g/100 g dry weight; else 0 points	0 to 1

**Table 2:** The Carbohydrate Food Quality Scoring System for CFQS-4 and CFQS-5 Models.

Models	Scoring System	Score Range	Higher Quality
CFQS-4:	1 point if fiber ≥ 10 g/100 g carb	0 to 4	4 points
	1 point if free sugar < 10 g/100 g carb		
	1 point if Na < 600 mg/100 g dry weight		
	1 point if K > 300 mg/100 g dry weight		
CFQS-5:	1 point if fiber ≥ 10 g/100 g carb	0 to 5	4 or 5 points **
	1 point if free sugar < 10 g/100 g carb		
	1 point if Na < 600 mg/100 g dry weight		
	1 point if K > 300 mg/100 g dry weight		
	1 point if whole grains ≥ 25 g/100 g dry weight		

** Higher quality CF defined by score of 4 or 5.

**Table 3:** Number and Percentage of “Higher-Quality Carbohydrate Foods” Based on Single Component and Composite Scoring Systems.

	Component Scores					Composite Scores		
	Fiber	Free Sugar	Sodium	Potassium	Whole Grain	(10:1:1)	(CFQS-4)	(CFQS-5)
Breads (*n* = 198)	60 (30.3%)	146 (73.7%)	22 (11.1%)	50 (25.3%)	65 (32.8%)	47 (23.7%)	2 (1.0%)	15 (7.6%)
Candy (*n* = 130)	11 (8.5%)	7 (5.4%)	129 (99.2%)	49 (37.7%)	0 (0%)	0 (0%)	0 (0%)	0 (0%)
Cooked Cereals (*n* = 111)	41 (36.9%)	65 (58.6%)	79 (71.2%)	82 (73.9%)	49 (44.1%)	29 (26.1%)	26 (23.4%)	39 (35.1%)
Cooked Grains (*n* = 55)	15 (27.3%)	54 (98.2%)	9 (16.4%)	13 (23.6%)	26 (47.3%)	15 (27.3%)	1 (1.8%)	7 (12.7%)
Crackers (*n* = 62)	19 (30.6%)	38 (61.3%)	21 (33.9%)	20 (32.3%)	20 (32.3%)	11 (17.7%)	7 (11.3%)	10 (15.1%)
Fruits (*n* = 117)	73 (62.4%)	83 (70.9%)	116 (99.1%)	111 (94.9%)	0 (0%)	62 (53.0%)	62 (53.0%)	62 (53.0%)
Other Desserts (*n* = 105)	3 (2.9%)	12 (11.4%)	94 (89.5%)	82 (78.1%)	0 (0%)	1 (1.0%)	1 (1.0%)	1 (1.0%)
Beans, Legumes (*n* = 92)	90 (97.8%)	86 (93.5%)	25 (27.2%)	90 (97.8%)	0 (0%)	85 (92.4%)	23 (25.0%)	23 (25.0%)
Quick Breads (*n* = 126)	7 (5.6%)	43 (34.1%)	34 (27.0%)	41 (32.5%)	27 (21.4%)	5 (4.0%)	1 (0.8%)	4 (3.2%)
Ready-to-Eat Cereals (*n* = 134)	56 (41.8%)	15 (11.2%)	103 (76.9%)	48 (35.8%)	97 (72.4%)	11 (8.2%)	10 (7.5%)	40 (29.9%)
Savory Snacks (*n* = 140)	35 (25.0%)	125 (89.3%)	76 (54.2%)	46 (32.6%)	43 (30.7%)	33 (23.6%)	6 (4.3%)	22 (15.6%)
Snack/Meal Bars (*n* = 46)	12 (26.1%)	0 (0%)	46 (100%)	23 (50.0%)	5 (10.9%)	0 (0%)	0 (0%)	0 (0%)
Sweet Bakery Products (*n* = 357)	10 (2.8%)	19 (5.3%)	308 (86.2%)	45 (12.4%)	5 (1.4%)	3 (0.8%)	0 (0%)	0 (0%)
Vegetables (*n* = 561)	420 (74.9%)	540 (96.2%)	159 (28.3%)	555 (98.9%)	0 (0%)	406 (72.4%)	124 (22.1%)	124 (22.1%)
Mixed dishes: grain (*n* = 362)	69 (19.1%)	349 (96.4%)	45 (12.4%)	235 (64.9%)	99 (27.3%)	69 (19.1%)	9 (2.5%)	50 (13.8%)
Total (*N* = 2596)	921 (36.51%)	1582 (60.9%)	1266 (48.8%)	1490 (57.4%)	436 (16.8%)	777 (29.9%)	272 (10.5%)	397 (15.3%)

Scores are represented in the table by both the number of foods and the percentage of foods in each category that meet the scoring criteria to be considered a “higher-quality carbohydrate food”. For the component scores, higher-quality carbohydrate foods are those which earn a single point. For the composite scoring systems, a food must earn 2 points to be considered higher-quality using the 10:1:1 metric, whereas it must earn 4 points in the CFQS-4 model, and either 4 or 5 points in the CFQS-5 model.

## Data Availability

Not applicable.

## Supplementary Materials

The following supporting information can be downloaded at: https://www.mdpi.com/article/10.3390/nu14071485/s1, Table S1: CFQS-4 and CFQS-5 Scores for 132 Carbohydrate Foods.
